# Neurobrucellosis and Multiple Sclerosis: Cause, Confounder, or Coincidence?

**DOI:** 10.1590/0037-8682-0154-2024

**Published:** 2025-09-22

**Authors:** Burak Kocaaga, Nermin Celik, Ozge Kaba, Melis Deniz, Nurhayat Yakut

**Affiliations:** 1 Basaksehir Cam and Sakura City Hospital, Department of Pediatrics, Division of Pediatric Infectious Diseases, Istanbul, Turkey.

**Keywords:** Brucellosis, Neurobrucellosis, Multiple sclerosis

## Abstract

We report the case of a 17-year-old girl who was initially diagnosed with multiple sclerosis based on clinical and radiological findings and later confirmed to have neurobrucellosis via cerebrospinal fluid Brucella polymerase chain reaction positivity. Magnetic resonance imaging revealed demyelinating lesions consistent with multiple sclerosis, and Brucella infection due to epidemiological exposure was suspected. To the best of our knowledge, this is the first pediatric report of coexisting neurobrucellosis and multiple sclerosis. It underscores the diagnostic challenges in distinguishing between infectious and autoimmune demyelinating disorders, particularly in endemic regions, and highlights the importance of a comprehensive evaluation of atypical presentations.

## INTRODUCTION

Brucellosis is a zoonotic infection caused by Gram-negative intracellular Brucella species. Although Brucella primarily affects livestock, it can also be transmitted to humans^1^. Eradication programs targeting animals have been implemented in many countries, but brucellosis remains endemic in several regions, particularly in the Mediterranean basin, including Türkiye, and often presents with nonspecific symptoms, making its diagnosis challenging^2^ ^,^ ^3^. Human brucellosis is a systemic zoonosis that can potentially affect multiple organ systems, including the hepatobiliary, musculoskeletal, genitourinary, and reticuloendothelial systems^1^. Among its diverse clinical presentations, neurobrucellosis is a rare but clinically significant form of central nervous system (CNS) involvement^1^ ^,^ ^2^.

The neurological spectrum encompasses meningitis, meningoencephalitis, cranial neuropathies, and demyelinating syndromes, which may mimic autoimmune demyelinating disorders such as multiple sclerosis (MS)^1^ ^-^ ^4^. Although MS typically presents in adolescents and young adults with multifocal demyelinating lesions, infectious etiologies, such as neurobrucellosis, must be considered in the differential diagnosis, particularly in endemic areas or when relevant epidemiological risk factors exist.

Here, we report the case of a 17-year-old girl who was initially diagnosed with MS based on clinical and radiological findings, but was later confirmed to have neurobrucellosis by cerebrospinal fluid (CSF) Brucella polymerase chain reaction (PCR) positivity. To our knowledge, this is the first reported pediatric case of coexisting features of multiple sclerosis and neurobrucellosis. This case highlights the diagnostic overlap between infectious and autoimmune demyelinating disorders and underscores the importance of a comprehensive evaluation before establishing a definitive diagnosis.

## CASE REPORT

**FIGURE 1:**
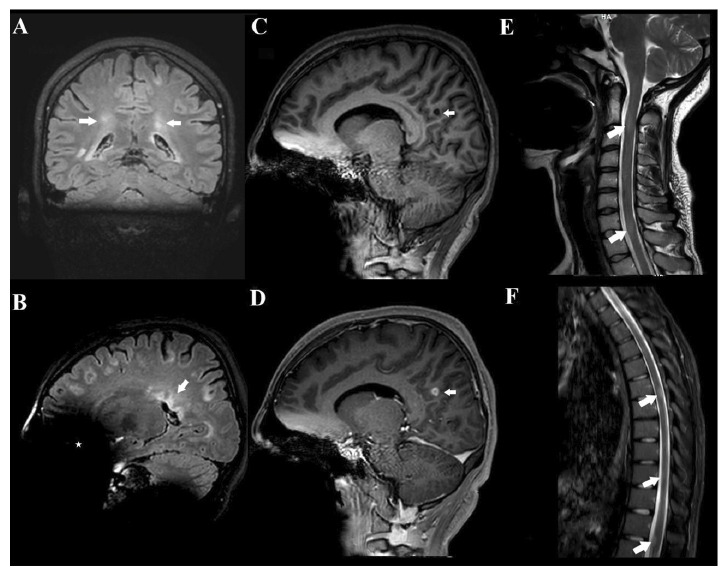
Pretreatment cranial MRI findings of the patient. **(A-B):** T2-weighted FLAIR MRI images revealed multiple demyelinating plaques in the bilateral frontoparietotemporal regions. The coronal image **(A):** shows hyperintense lesions (arrows) in both juxtacortical and deep white matter, oriented perpendicular to the lateral ventricles. The sagittal image **(B):** demonstrates the characteristic “Dawson’s fingers” (arrow), indicative of perivenular demyelination. An asterisk (*) marks an artifact caused by the patient's dental braces. **(C-D):** Sagittal T1-weighted images before and after contrast administration demonstrate no enhancement on the precontrast image **(C, arrow)**, while the post-contrast image **(D, arrow)** reveals a 10 mm enhancing lesion consistent with active demyelination following intravenous gadolinium administration. **(E-F):** Demyelinating plaques at the cervical (**arrows in E**) and thoracic (**arrows in F**) levels.

**FIGURE 2:**
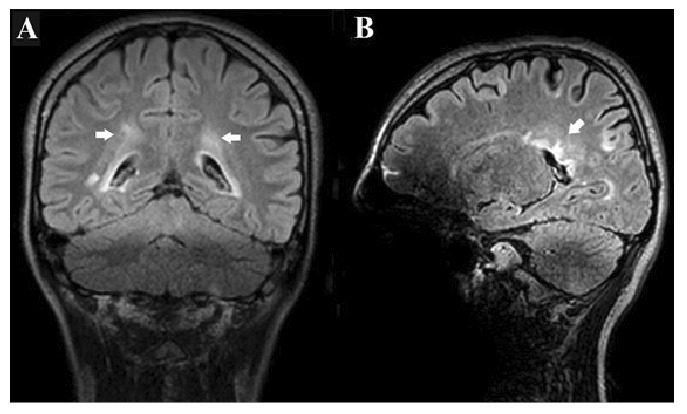
**(A-B):** Post-treatment images obtained after 2 weeks of antimicrobial therapy show a subtle reduction in the size and signal intensity of the previously observed periventricular white matter lesions, suggesting mild radiological improvement.

**FIGURE 3:**
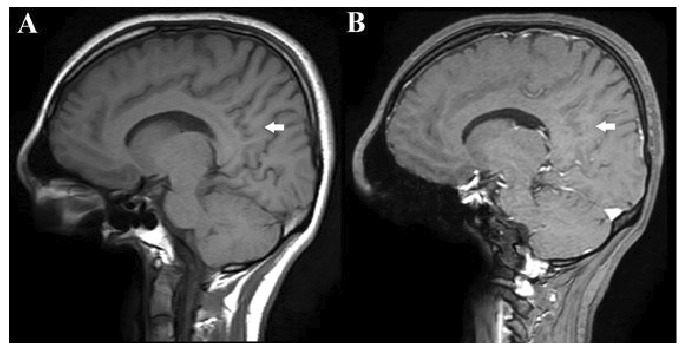
Follow-up sagittal T1-weighted images obtained after 1 month of antimicrobial therapy. The precontrast image **(A):** shows no visible lesion, and the post-contrast image **(B):** demonstrates no enhancement at the site of the previously observed lesion **(arrow)**, indicating resolution of contrast uptake and radiological improvement.

A 17-year-old girl presented with a 1-year history of recurrent dizziness and vertigo. Recently, her symptoms had worsened, with newly developed blurred vision and numbness of the face and extremities. Neurological examination results were unremarkable, with no signs of meningeal irritation. No history of raw milk consumption was reported. However, the patient occasionally consumed traditionally prepared cheese, which is a potential risk factor for brucellosis in endemic areas. Her family history included brucellosis in the grandmother and MS in a friend. Initial laboratory evaluation revealed normal white blood cell count (5.78 ×10⁹/L), neutrophils (3.19 ×10⁹/L), lymphocytes (2.08 ×10⁹/L), hemoglobin (11.9 g/dL), and platelet count (323 ×10⁹/L). C-reactive protein (2.7 mg/L), procalcitonin (0.04 ng/mL), and erythrocyte sedimentation rate were all within normal limits. Brain magnetic resonance imaging (MRI) revealed demyelinating plaques in the bilateral frontoparietal regions and a contrast-enhancing active lesion in the left parietal lobe (Figure 1A-D). Spinal MRI revealed demyelinating plaques at the cervical and thoracic levels (Figure 1E-F). The patient was initially diagnosed with MS. Lumbar puncture revealed clear acellular CSF with normal opening pressure. CSF analysis showed a protein level of 17.6 mg/dL and a glucose level of 63 mg/dL, with a concurrent serum glucose level of 85 mg/dL. The meningitis/encephalitis PCR panel was negative, and the cultures remained sterile. Oligoclonal bands were detected in the CSF. High-dose corticosteroid therapy was initiated, but discontinued on day five after CSF Brucella PCR positivity. The patient was diagnosed with neurobrucellosis and treated with ceftriaxone, rifampicin, and doxycycline. During the second week of treatment, the patient developed hand tremors, prompting a repeat cranial MRI. Lesions identified in prior coronal images, hyperintense areas in both the juxtacortical and deep white matter oriented perpendicular to the lateral ventricles, appeared slightly smaller on follow-up imaging (Figure 2A). Sagittal images also showed slight regression of periventricular demyelination (Figure 2B). No clear etiology was found for the tremor, which resolved spontaneously within 48 h and did not recur. Neurobrucellosis symptoms improved with steroids and were fully resolved after 1 month of ceftriaxone-based therapy. Following a 1-month course of treatment with ceftriaxone, rifampicin, and doxycycline, a follow-up lumbar puncture was performed, which revealed normal CSF findings and a negative Brucella PCR result. Follow-up brain MRI revealed radiological improvements. The previously observed contrast-enhancing demyelinating plaque located parasagittally in the left parietal centrum semiovale resolved (Figure 3A,B). Following completion of the initial 1-month intravenous ceftriaxone therapy, the patient was discharged and continued on oral rifampicin and doxycycline, with treatment planned to be completed over at least a 12-week course. Outpatient follow-ups with an infectious disease team are ongoing, and the neurology department continues to monitor MS-specific conditions.

## DISCUSSION

Neurobrucellosis may present with various cranial MRI findings, although a substantial number of patients show normal findings. When present, abnormalities typically reflect CNS inflammation, white matter changes, and vascular involvement. Inflammatory findings may appear as diffuse leptomeningeal enhancement or localized changes such as cranial nerve involvement, granulomas, or arachnoiditis. White matter lesions often present as T2 hyperintensities in the arcuate fibers, periventricular regions, or focal demyelinating areas. Vascular pathology may include lacunar infarction, microhemorrhage, or venous thrombosis^5^.

In contrast to the typical imaging patterns associated with neurobrucellosis, our patient exhibited radiological features consistent with MS. MRI revealed periventricular white matter plaques, juxtacortical lesions perpendicular to the lateral ventricles, and Dawson’s fingers, which are classic hallmarks of demyelination in MS^6^. Meningeal enhancement, cranial nerve involvement, or vascular abnormalities were not observed. While the MRI findings of our patient supported the diagnosis of multiple sclerosis, the detection of Brucella DNA in the CSF confirmed neurobrucellosis, indicating the possible coexistence of these two distinct diseases.

Neurobrucellosis may mimic various neurological disorders including MS^3^. Several cases have described MS-like presentations that were later identified as neurobrucellosis^7^ ^-^ ^9^. Infections are also thought to trigger autoimmune processes, including MS^10^ ^,^ ^11^. A literature review revealed several pediatric cases in which neurobrucellosis clinically mimicked demyelinating diseases; however, no previously reported pediatric case has confirmed the coexistence of neurobrucellosis and MS in the same patient^4^. In this patient, a positive Brucella PCR test result for CSF and oligoclonal bands suggested a possible infection-induced autoimmune response, although their coexistence may be coincidental. This case highlights the clinical and radiological overlap between neurobrucellosis and MS, underscoring the importance of considering infectious etiologies in the differential diagnosis.

## Data Availability

Research data is only available upon request.
