# Erratum

**DOI:** 10.1590/0037-8682-0154B-2024

**Published:** 2025-10-03

**Neurobrucellosis and Multiple Sclerosis: Cause, Confounder, or Coincidence?**

 In the document: Kocaaga B, Celik N, Kaba O, Deniz M, Yakut N. Neurobrucellosis and Multiple Sclerosis: Cause, Confounder, or Coincidence?. **Rev Soc Bras Med Trop Internet. 2025;58:e0154-2025**. Available from: https://doi.org//10.1590/0037-8682-0154-2024 

**CASE REPORT - Page 1**

 Her family history included brucellosis in the grandmother and **MS in a friend**. 

**Is correct as:**

 Her family history included brucellosis in the grandmother and **MS in a cousin.**

